# Supplementary material for: Geographic range of plants drives long-term climate change
Source: Nat Commun. 2024 Feb 28;15:1805. doi: 10.1038/s41467-024-46105-1 (PMC10901853; doi:10.1038/s41467-024-46105-1)
Supplement: Supplementary file 1 — Supplementary Information [file 41467_2024_46105_MOESM1_ESM.pdf]

Supplementary Information

Geographic range of plants drives long-term climate change

Khushboo Gurung[1]*, Katie J. Field[2], Sarah A. Batterman[3,4,5], Simon W. Poulton[1], Benjamin J. W. Mills[1]

[1]School of Earth and Environment, University of Leeds, Leeds LS2 9JT, UK

[2]Plants, Photosynthesis and Soil, School of Biosciences, University of Sheffield, Sheffield, S10 2TN UK

[3]Cary Institute of Ecosystem Studies, Millbrook, NY 12545, USA

[4]School of Geography, University of Leeds, Leeds LS2 9JT, UK

[5]Smithsonian Tropical Research Institute, Panama City, Panama, USA

*Corresponding author: k.gurung@leeds.ac.uk

Comparison of present-day and modelled biomass

Mirroring the analysis of ref[1] we compare SCION-FLORA modelled present-day biomass to the European Space Agency (ESA) dataset[2] (Fig. S1). Despite the low resolution climate model, and use of a fast vegetation model, the general latitudinal and longitudinal patterns of vegetation are clearly reproduced. The largest discrepancy is observed around the equator and in the high northern latitudes. The higher equatorial discrepancy is driven by very high biomass in the equator in general, and the high latitude discrepancy is partly due to the absence of biomass in very high latitudes in SCION-FLORA, which is a result of the ice parameter (Fig. S2) that dictates land with temperatures < -10°C as ice and therefore uninhabitable for plants. The model also does not contain grasses and tundra species, which may increase these mismatches. The vegetation model also simplifies more complicated interactions such as soil-related nutrient exchange involving mycorrhizas[3]. Future iterations of this research should include a more detailed representation of larger ecosystem interactions in order to capture the impact of biosphere on climate.

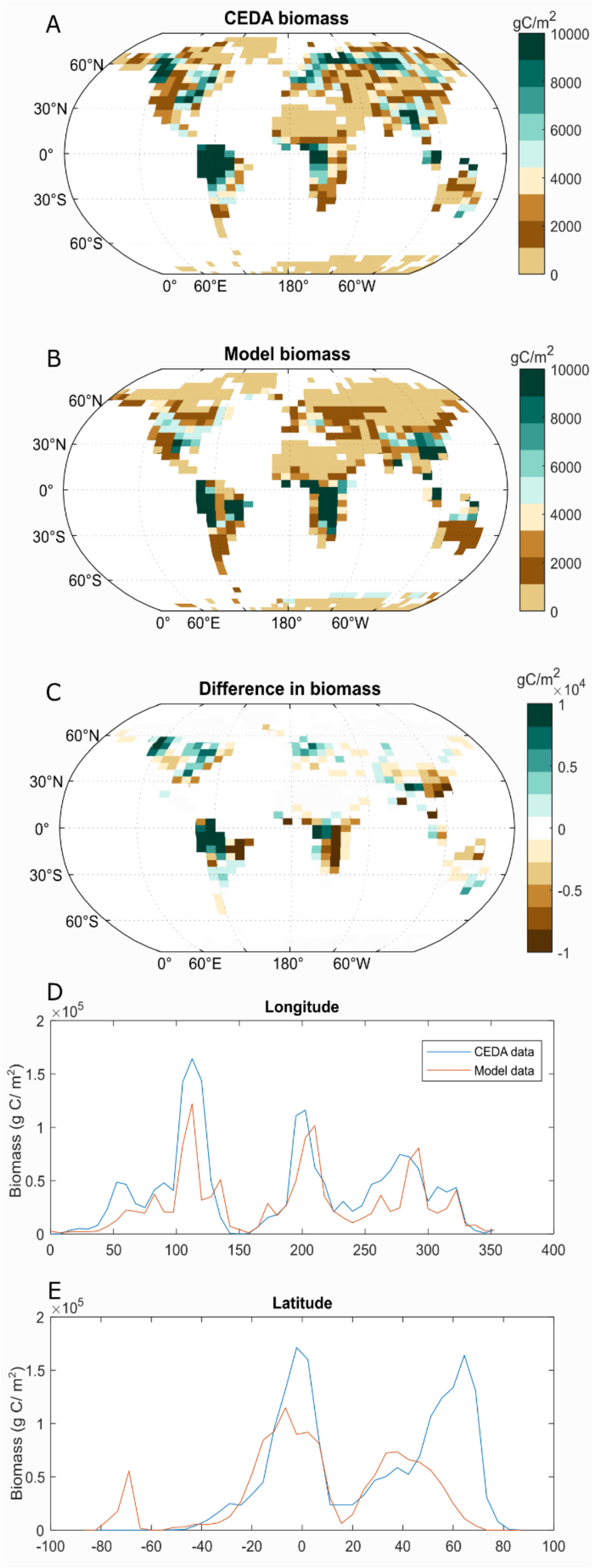

Figure S1. Comparison between CEDA and modelled biomass. **(A)** Centre for Environmental Data Analysis (CEDA) biomass[2] scaled down to model output resolution. **(B)** Modelled biomass produced by FLORA using data from ref[1]. **(C)** Difference in biomass between CEDA and model output highlighting areas of over- and under-prediction. **(D,E)** Longitudinal and latitudinal comparison between CEDA and modelled biomass. All datasets can be found in the Data folder on Github.

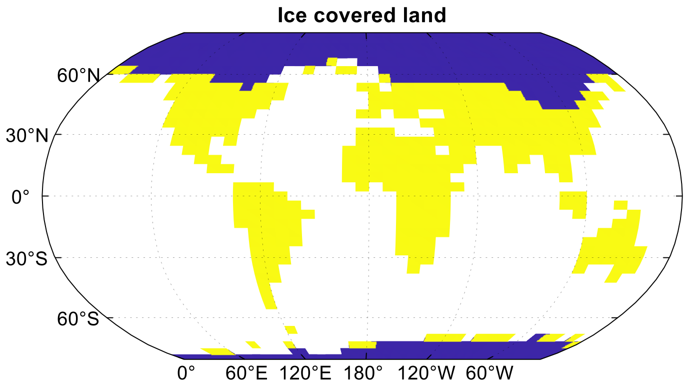

Figure S2. Ice mask with the present day geographical configuration based on FOAM projections.

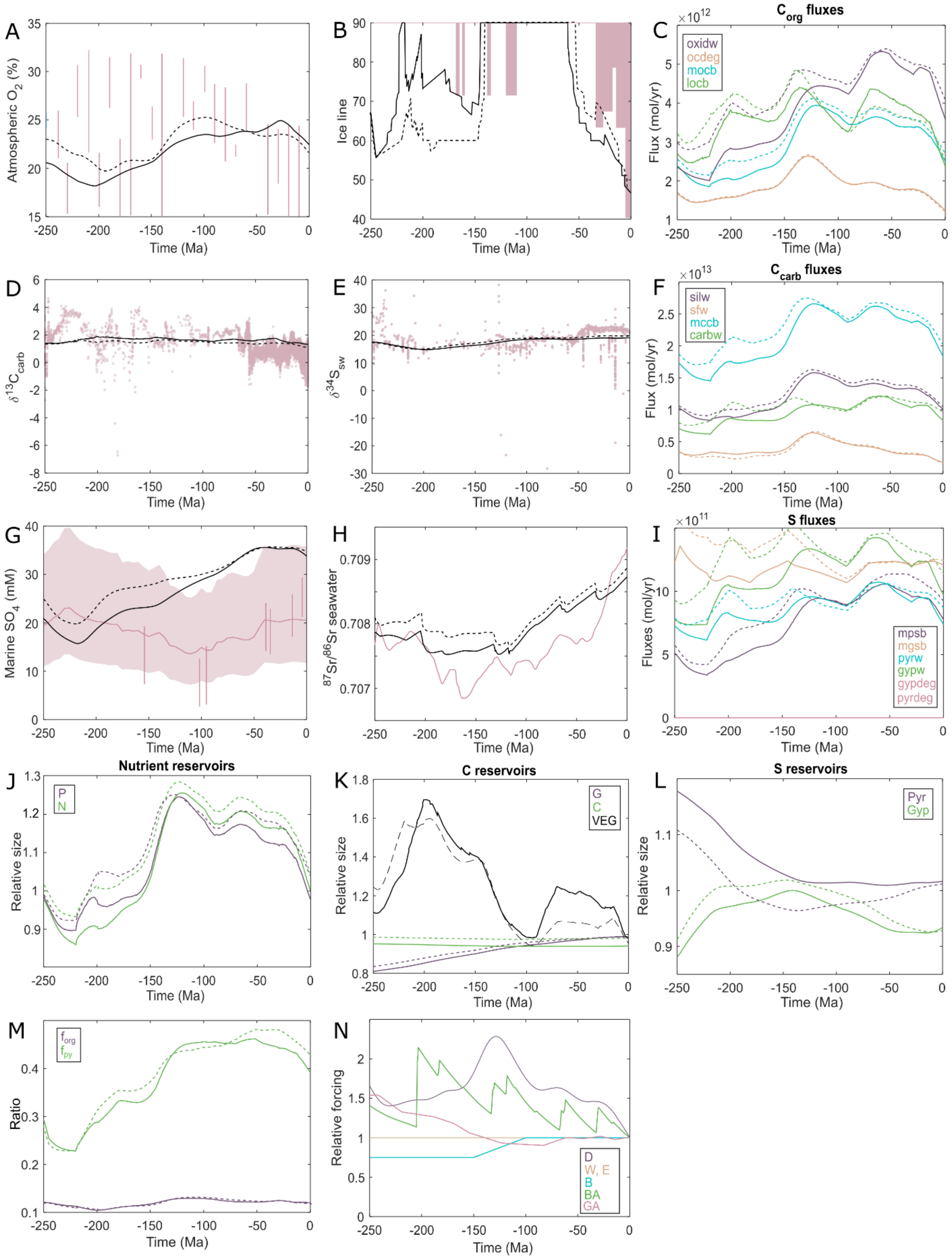

Figure S3. Model outputs and fluxes over time from SCION-FLORA (black or coloured solid lines), SCION (black or coloured dotted lines) and proxy data (pink). **(A)** Atmospheric O_2 prediction compared to the charcoal record[4]; **(B)** Ice line latitude plotted against the geological record[5]; **(C)** Organic carbon fluxes (oxidw: organic carbon weathering, ocdeg: organic carbon degassing, mocb: marine organic carbon burial, locb: land organic carbon); **(D)** Carbonate $\delta^{13}C$ estimates compared to the geological

record[6]; **(E)** Seawater δ^{34}S compared to the geological record of evaporites, barite and carbonate associated sulfate (CAS)[7]; **(F)** Carbonate fluxes (silw: silicate weathering, sfw: seafloor weathering, mccb: carbonate burial, carbw: carbonate weathering); **(G)** Marine SO_4 concentrations compared to fluid inclusion data[8,9,10]; **(H)** Seawater strontium ^{87}S/^{86}S ratio compared to the geological record[11]; **(I)** Sulfur fluxes (mpsb: pyrite burial, mgsb: gypsum burial, pyrw: pyrite weathering, gypw: gypsum weathering, gypdeg: gypsum degassing*, pyrdeg: pyrite degassing*); **(J)** Relative ocean nitrate (N) and phosphate (P) reservoir size; **(K)** Relative carbon reservoirs (G: buried organic carbon, C: buried carbonate carbon, VEG: vegetation biomass); **(L)** Relative sulfur reservoirs (Pyr: buried pyrite sulfur, Gyp: buried gypsum sulfur); **(M)** Ratio of organic carbon (f_{org}) and pyrite (f_{py}) fluxes; **(N)** Relative forcings (D: degassing, W: biotic weathering*, E: plant evolution*, B: burial depth, BA: basalt area, GA: granite area). *lines overlap.

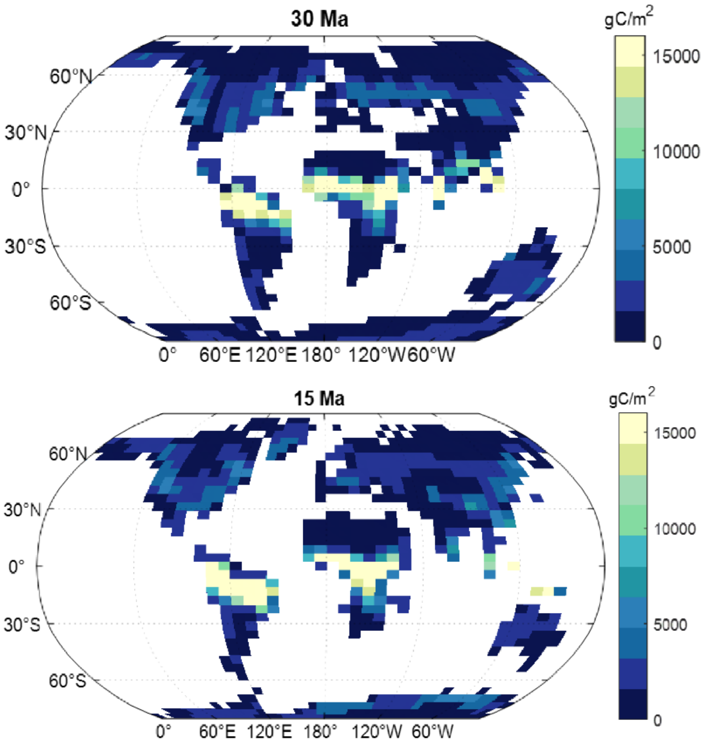

Figure S4. Modelled biomass maps for 30 and 15 million years ago (Ma). Despite the increased intensity of biomass across the tropics at 15 Ma, changes in paleogeography led to the disappearance of parts of Australia, South East Asia and Africa.

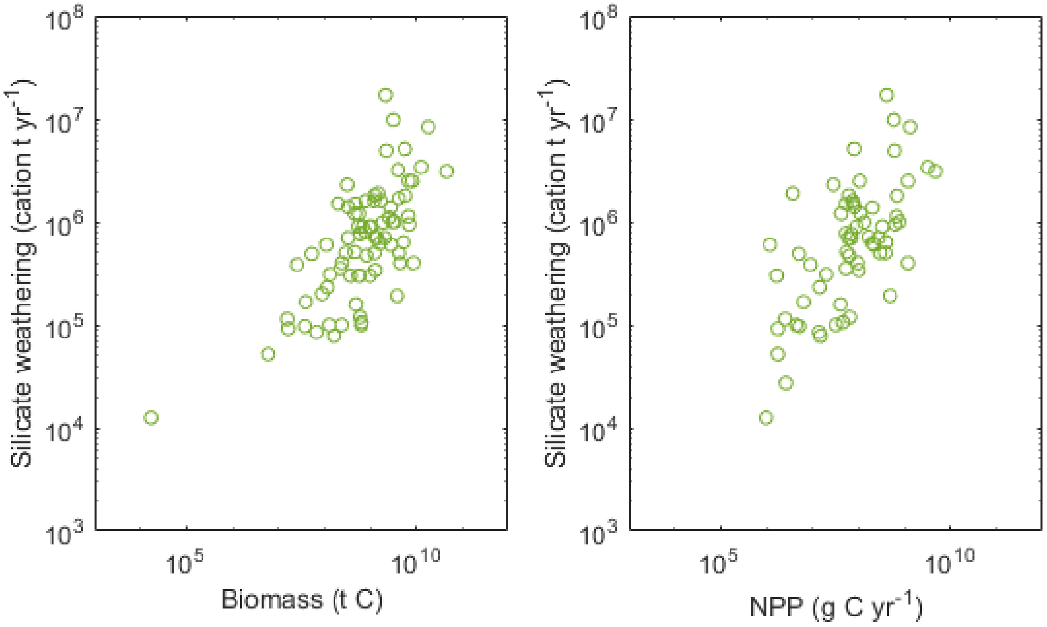

Figure S5. Relationship between NPP, biomass and silicate weathering. Relationship between river basin silicate weathering and catchment biomass (left), catchment NPP (right). Catchment maps are from ref 12, river catchment data are from ref 13, biomass and NPP data from CDIAC[14] and NASA EOS[15] databases, respectively. NPP: Net primary productivity.

Supplementary References

1. Gurung, K., Field, K.J., Batterman, S.A., Godderis, Y., Donnadieu, Y., Porada, P., Taylor ,L.L., Mills, B.J.W. Climate windows of opportunity for plant expansion during the Phanerozoic. *Nat. Comms* **13**, (2022).
2. Santoro, M.; Cartus, O. ESA Biomass Climate Change Initiative (Biomass_cci): Global datasets of forest above-ground biomass for the year 2017, v1. Centre for Environmental Data Analysis. (2019).
3. Hawkins, H-J., Cargill, R.I.M., Nuland, M.E.V., Hagen, S.C., Field, K.J., Sheldrake, M., Soudzilovskaia, N.A., Kiers, E.T. Mycorrhizal mycelium as a global carbon pool. *Curr. Biol.* **11**, (2023).
4. Glasspool I.J., Scott, A.C. Phanerozoic concentration of atmospheric oxygen reconstructed from sedimentary charcoal. *Nat. Geosci.* **3**, 627-630 (2010).
5. Cather A.M., Dunbar, N.W., McDowell, F.W., McIntosh, W.C., Scholle, P.A. Climate forcing by iron fertilization from repeated ignimbrite eruptions: The icehouse-silicic large igneous province (SLIP) hypothesis. *Geosphere* **5**, 315-324 (2009).
6. Saltzman, M.R., Thomas, E. Carbon Isotope Stratigraphy pp. 207-232 (2012).
7. Crockford, P.W., Kunzmann, M., Bekker, A., Hayles, J., Bao, H., Halverson, G.P., Peng, Y., Bui, T.H., Cox, G.M., Gibson, T.M., Worndle, S., Rainbird, R., Lepland, A., Swanson-Hysell, N.L., Master, S., Sreenivas, B., Kuznetsov, A., Krupenik, V., Wing, B.A. Claypool continued: Extending the isotopic record of sedimentary sulfate. *Chem. Geol.* **513**, 200-225 (2019).

8. Horita, J., Zimmermann, H., Holland, H.D. Chemical evolution of seawater during the Phanerozoic: Implications from the record of marine evaporates. *Geochim. Cosmochim. Acta* **66**, 3733-3756 (2002).
9. Brennan, S.T., Lowenstein, T.K., Horita, J. Seawater chemistry and the advent of biocalcification. *Geology* **32**, 473-476 (2004).
10. Lowenstein, T.K., Timofeeff, M.N., Kovalevych V.M., Horita, J. The major-ion composition of Permian seawater. *Geochim. Cosmochim.* Acta **69**, 1701-1719 (2005).
11. McArthur, J.M., Howarth, R.J., Shields, G.A. Strontium Isotope Stratigraphy pp 127-144 (2012).
12. Maffre, P., Ladant, J.B., Moquet, J.S., Carretier, S., Labat, D., Godderis, Y. Mountain ranges, climate and weathering. Do orogens strengthen or weaken the silicate weathering carbon sink? *Earth and Planetary Science Letters* **493**, 174-185 (2018).
13. Gaillardet, J., Dupre, B., Louvat, P., Allegre, C.J. Global silicate weathering and CO_2 consumption rates deduced from the chemistry of large rivers. *Chemical geology* **159**, 3-30 (1999).
14. Ruesch, A., Holly, K.G. New IPCC Tier-1 Global biomass carbon map for the Year 2000 (2008).
15. Running, S., Zhao, M. MODIS/Terra Net Primary Production Gap-Filled Yearly L4 Global 500m SIN Grid V061. NASA EOSDIS Land processes distributed active archine centre. https://doi.org/10/5067/MODIS/MOD17A3HGF.061 (2021).
